# Subspecies Distribution and Antimicrobial Susceptibility Testing of Mycobacterium abscessus Clinical Isolates in Madrid, Spain: a Retrospective Multicenter Study

**DOI:** 10.1128/spectrum.05041-22

**Published:** 2023-05-22

**Authors:** Alba Ruedas-López, Marta Tato, Antonio Broncano-Lavado, Jaime Esteban, María Jesús Ruiz-Serrano, María Sánchez-Cueto, Carlos Toro, Diego Domingo, Juana Cacho, Laura Barrado, Paula López-Roa

**Affiliations:** a Clinical Microbiology and Parasitology Department, Instituto de Investigación, Hospital Universitario, Madrid, Spain.; b Clinical Microbiology and Parasitology Department, Hospital Universitario Ramón y Cajal, Madrid, Spain; c Clinical Microbiology and Parasitology Department, Hospital Universitario Fundación Jiménez Díaz, Madrid, Spain; d Centro de Investigación Biomédica en Red (CIBER) de Enfermedades infecciosas CIBERINFEC, Spain; e Clinical Microbiology and Infectious Diseases, Hospital General Universitario Gregorio Marañón, Madrid, Spain.; f Clinical Microbiology and Infectious Diseases, Instituto de Investigación Sanitaria Gregorio Marañón (IiSGM) Madrid, Madrid, Spain; g Centro de Investigación Biomédica en Red (CIBER) de Enfermedades Respiratorias- CIBERES (CB06/06/0058), Madrid, Spain; h Clinical Microbiology and Parasitology Department, Hospital Universitario La Paz, Madrid, Spain; i Clinical Microbiology and Parasitology Department, Hospital Universitario de La Princesa, Madrid, Spain; j Clinical Microbiology and Parasitology Department, Hospital Universitario de Getafe, Madrid, Spain; k Clinical Microbiology and Parasitology Department, Hospital Universitario de Móstoles, Madrid, Spain; Quest Diagnostics Nichols Institute

**Keywords:** *Mycobacterium abscessus*, subspecies, GenoType, antimicrobial susceptibility, epidemiology, inducible resistance, *in vitro* susceptibility, subspecies distribution

## Abstract

Mycobacterium abscessus (MABS) is the most pathogenic and drug-resistant rapidly growing mycobacteria. However, studies on MABS epidemiology, especially those focusing on subspecies level, are scarce. We aimed to determine MABS subspecies distribution and its correlation with phenotypic and genotypic antibiotic profiles. A retrospective multicenter study of 96 clinical MABS isolates in Madrid between 2016 to 2021 was conducted. Identification at the subspecies level and resistance to macrolides and aminoglycosides were performed by the GenoType NTM-DR assay. The MICs of 11 antimicrobials tested against MABS isolates were determined using the broth microdilution method (RAPMYCOI Sensititer titration plates). Clinical isolates included 50 (52.1%) MABS subsp. *abscessus*; 33 (34.4%) MABS subsp. *massiliense*; and 13 (13.5%) MABS subsp. *bolletii*. The lowest resistance rates corresponded to amikacin (2.1%), linezolid (6.3%), cefoxitin (7.3%), and imipenem (14.6%), and the highest to doxycycline (100.0%), ciprofloxacin (89.6%), moxifloxacin (82.3%), cotrimoxazole (82.3%), tobramycin (81.3%), and clarithromycin (50.0% at day 14 of incubation). Regarding tigecycline, although there are no susceptibility breakpoints, all strains but one showed MICs ≤ 1 μg/mL. Four isolates harbored mutations at positions 2058/9 of the *rrl* gene, one strain harbored a mutation at position 1408 of the *rrl* gene, and 18/50 harbored the T28C substitution at *erm*(41) gene. Agreement of the GenoType results with clarithromycin and amikacin susceptibility testing was 99.0% (95/96). The rate of MABS isolates showed an upward trend during the study period, being M. abscessus *subsp. abscessus* the most frequently isolated subspecies. Amikacin, cefoxitin, linezolid, and imipenem showed great *in vitro* activity. The GenoType NTM-DR assay provides a reliable and complementary tool to broth microdilution for drug resistance detection.

**IMPORTANCE** Infections caused by Mycobacterium abscessus (MABS) are increasingly being reported worldwide. Identifying MABS subspecies and assessing their phenotypic resistance profiles are crucial for optimal management and better patient outcomes. *M. abscessus* subspecies differ in *erm*(41) gene functionality, which is a critical determinant of macrolide resistance. Additionally, resistance profiles of MABS and the subspecies distribution can vary geographically, highlighting the importance of understanding local epidemiology and resistance patterns. This study provides valuable insights into the epidemiology and resistance patterns of MABS and its subspecies in Madrid. Elevated resistance rates were observed for several recommended antimicrobials, emphasizing the need for cautious drug use. Furthermore, we assessed the GenoType NTM-DR assay, which examines principal mutations in macrolides and aminoglycosides resistance-related genes. We observed a high level of agreement between the GenoType NTM-DR assay and the microdilution method, indicating its usefulness as an initial tool for early initiation of appropriate therapy.

## INTRODUCTION

Mycobacterium abscessus complex (MABC) is a group of rapidly growing, multidrug-resistant non-tuberculous mycobacteria (NTM) responsible for progressive pulmonary disease and soft tissue and wound infections. Over the last 2 decades, pulmonary infections caused by Mycobacterium abscessus have increased, especially in patients with cystic fibrosis (1, 2). In a recent multicenter study in Madrid, we observed an increased prevalence of NTM isolates with a 2-fold increase regarding the M. abscessus group (3). This finding led us to conduct the present study focusing on the epidemiology of this worrisome pathogen in our region.

M. abscessus has undergone multiple taxonomic changes since its first classification (4–7). Although controversy about its taxonomy remains (8–10), the most widespread nomenclature is Mycobacterium abscessus (MABS) that comprises 3 subspecies: M. abscessus *subsp. abscessus* (MABSa), M. abscessus *subsp. bolletii* (MABSb), and M. abscessus *subsp. massiliense* (MABSm) (11). Importantly, these 3 subspecies differ in macrolide susceptibility based on the presence and functional status of the *erythromycin ribosomal methylation* gene *41* (*erm41*). In MABSa, a T/C polymorphism at position 28 of the *erm*(41) sequence determines the presence (28T) or absence (T28C) of inducible macrolide resistance. Although most of MABSa and MABSb strains show inducible macrolide resistance, those isolates that harbor the T28C *erm*(41) substitution become macrolide-sensitive in the absence of other resistance mechanisms due to loss of functionality of the *erm*(41) gene. MABSm harbors a large deletion in the *erm*(41) gene, rendering the gene nonfunctional and the mycobacteria susceptible to macrolides (12, 13), which might explain the more favorable clinical outcomes upon clarithromycin treatment (14). Since extended periods of incubation are required to phenotypically detect inducible antibiotic profiles, molecular methods targeting the *erm*(41) gene have emerged as an alternative tool to phenotypic testing.

M. abscessus also exhibits acquired resistance to macrolides and aminoglycosides. Due to the prolonged, repeated, or inappropriate use of these antibiotics, M. abscessus isolates with acquired macrolide and aminoglycoside resistance can emerge, primarily due to modifications of the genes encoding the antibiotic targets (*rrs* and *rrl*, respectively) (15–17).

In regard to its multi-resistant nature, antibiotic susceptibility testing, including determination of the *erm*(41) genotype, is recommended before starting treatment and after each recurrent episode of MABS infection (18). Knowledge of the local epidemiology, including subspecies distribution and susceptibility profiles, can be helpful for the initial management of these patients while waiting for further subspecies identification and resistance profile. However, there is very limited information about the local epidemiology of MABS in our region.

The aims of this study were to assess the subspecies distribution of MABS clinical isolates during the study period in seven tertiary care hospitals in Madrid, to evaluate the presence of macrolide and aminoglycosides resistance-related genes of these isolates, and to determine the level of agreement between phenotypic and genotypic resistance profile.

## RESULTS

**FIG 1:**
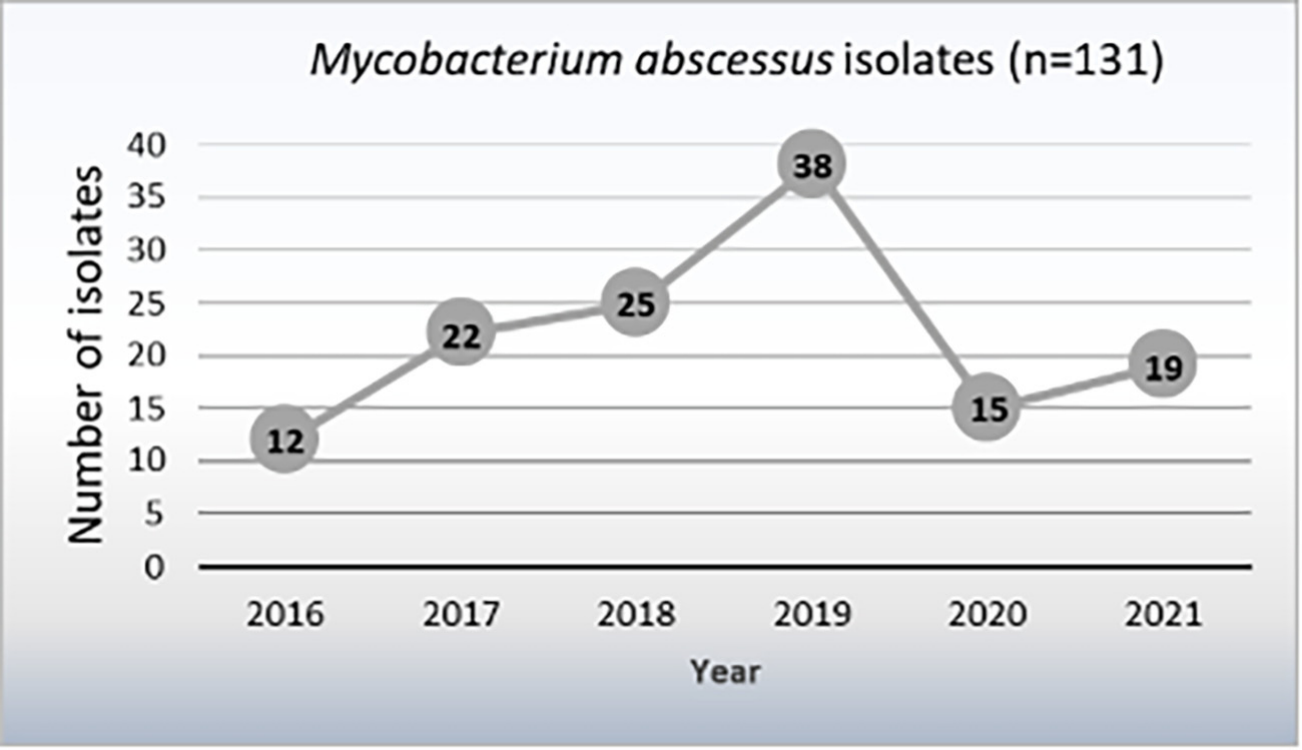
Time distribution of Mycobacterium abscessus clinical isolates obtained from 131 patients over period of 6 years. Only the first isolation of each patient was included.

We identified 375 M. abscessus isolates from 131 patients. Only the first isolation of each patient was included in the study. There were 35 isolates that could not be recovered because they were not stored in a strain collection or because the strains were not viable after seeding. The analysis of the demographic data will be shown for the total of all 131 patients, whereas the phenotypic and genotypic analyses will be shown for the total of the 96 viable strains. Among the 131 included patients, 77 were women (58.8%). The mean age was 53.1 (SD = 21.9) and 53.9 (SD = 24.8) years for women and men, respectively. Regarding nationality, most patients were Spanish (76.3%), followed by South American (10.7%), 10.7% had an unknown origin, and to a lesser proportion (2.3%) were Moroccan, Chinese, and Equatoguinean. The majority of the MABS strains were isolated from respiratory specimens (118/131; 90.1%), followed by skin and soft tissues (10/131, 7.6%); and blood (3/131, 2.3%). The largest number of strains were isolated in 2019 and the lowest in 2016. Time distribution of MABS isolates is showed in Fig. 1.

**FIG 2:**
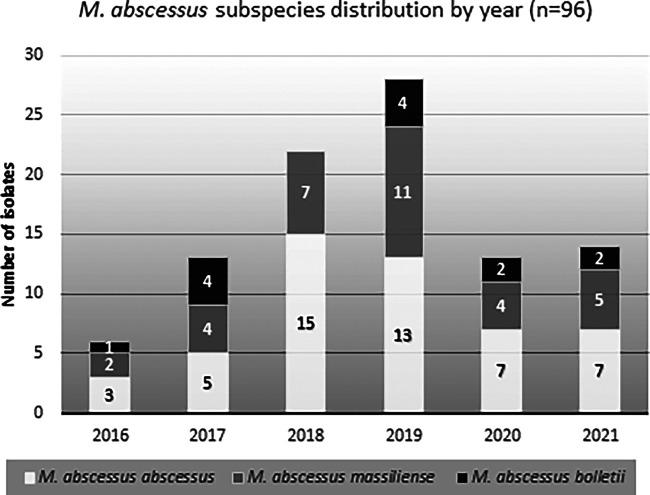
Time distribution of Mycobacterium abscessus subspecies over a 6-year period, based on 96 viable strains using the GenoType assay.

Out of 96 MABS clinical isolates, 50 (52.1%) were identified as MABSa, 33 (34.4%) as MABSm and 13 (13.5%) as MABSb. Fig. 2 shows the number of each subspecies ranked by year.

### Phenotypic susceptibility testing.

Distribution and interpretation of MICs according to subspecies identification is summarized in Table 1. All isolates were resistant to doxycycline, with MICs > 16 μg/mL (data not shown).

**TABLE 1:** Antimicrobial susceptibility testing of Mycobacterium abscessus^*a*^

Drug subsp.	No. of strains at each MIC (μg/mL)													N (%) resistant
	≤ 0,06	0,12	0,25	0,5	1	2	4	8	16	32	64	128	> 128	
Cotrimoxazole														
MABSa (*n* = 50)				1		5	4^*a*^	4^*b*^	36^*a*^^*c*^					40 (80.0)
MABSb (*n* = 13)								1^*b*^	12^*b*^^*c*^					13 (100.0)
MABSm (*n* = 33)					1	3	3^*a*^	4^*b*^	22^*b*^^*c*^					26 (78.8)
Ciprofloxacin														
MABSa						4^*a*^	21^*b*^	25^*b*^^*c*^						46 (92.0)
MABSb							5^*b*^	8^*b*^^*c*^						13 (100.0)
MABSm					1	5^*a*^	5^*b*^	22^*b*^^*c*^						27 (81.8)
Moxifloxacin														
MABSa					1	11^*a*^	12^*b*^	14^*b*^	12^*b*^^*c*^					38 (76.0)
MABSb							5^*b*^	3^*b*^	5^*b*^^*c*^					13 (100.0)
MABSm				1	1	3^*a*^	11^*b*^	3^*b*^	14^*b*^^*c*^					28 (84.8)
Cefoxitin														
MABSa								1	25	13^*a*^	7^*a*^	3^*b*^	1^*b*^	4 (8.0)
MABSb									6	7^*a*^				0 (0.0)
MABSm									10	14^*a*^	6^*a*^	3^*b*^		3 (9.1)
Tigecycline^*d*^														
MABSa	*5*	*19*	*15*	*9*	*1*			*1*						*^d^*
MABSb		*6*	*6*	*1*										*^d^*
MABSm	*4*	*7*	*12*	*10*										*^d^*
Amikacin														
MABSa						3	25	14	7			1^*b*^^*c*^		1 (2.0)
MABSb							5	5	3					0 (0.0)
MABSm				1^*e*^		5	14	9	3			1^*b*^^*c*^^*f*^		1 (3.0)
**Drug subsp.**	**No. of strains at each MIC (μg/mL)**													
	**≤ 0,06**	**0,12**	**0,25**	**0,5**	**1**	**2**	**4**	**8**	**16**	**32**	**64**	**128**	**> 128**	
Clarithromycin (3 days)														
MABSa	13	10	8	11	6	1				1^*b*^^*c*^^*g*^				1 (2.0)
MABSb	1	5	1	4	2									0 (0.0)
MABSm	11	11	6	2						3^*b*^^*c*^^*g*^				3 (9.1)
Clarithromycin (14 days)^*h*^														
MABSa	1^*i*^	6^*i*^	1^*i*^	2^*i*^	7^*i*^	1^*i*^		2		29^*b*^^*c*^				31 (63.3)^*h*^
MABSb										13^*b*^^*c*^				13(100.0)^*h*^
MABSm		13	6	7	3		1							0 (0.0)^*h*^
Linezolid														
MABSa						6	12	15	12^*a*^	2^*b*^	3^*b*^^*c*^			5 (10.0)
MABSb							2	7	4^*a*^					0 (0.0)
MABSm				2^*e*^		4	5	13	8^*a*^	1^*b*^				1 (3.0)
Imipenem	
MABSa							12	14^*a*^	19^*a*^	2^*b*^	2^*b*^	1^*b*^^*c*^		5 (10.0)
MABSb							4	6^*a*^	3^*a*^					0 (0.0)
MABSm							5	9^*a*^	10^*a*^	5^*b*^	3^*b*^	1^*b*^^*c*^		9 (27.3)
Tobramycin	
MABSa						1	16^*a*^	20^*b*^	11^*b*^	2^*b*^^*c*^				33 (66.0)
MABSb							1^*a*^	4^*b*^	7^*b*^	1^*b*^^*c*^				12 (92.3)
MABSm							3^*a*^	16^*b*^	14^*b*^					30 (90.9)

a Strains with MICs interpreted as susceptible with increased exposure.

b Strains with MICs interpreted as resistant.

c Off-scale high MICs were included at the next highest concentration.

d There are no breakpoints to tigecycline. Number of strains showed in italic type.

e Off-scale low MICs were included at the previous lowest concentration.

f Strains harboring a *rrs* mutation.

g Strains harboring a *rrl* mutation.

h Strains showing acquired resistance to clarithromycin excluded (MABSa *n* = 49; MABSb *n* = 13, MABSm *n* = 30).

i Strains harboring the T28C mutation at *erm(41)*.

The most resistant *in vitro* antimicrobials against MABS isolates were doxycycline (100.0%), ciprofloxacin (89.6%), moxifloxacin (82.3%), cotrimoxazole (82.3%), and tobramycin (81.3%). Isolates of MABS showed low resistance rates to amikacin (2.1%), linezolid (6.3%), cefoxitin (7.3%), and imipenem (14.6%). Forty-eight isolates (50.0%) were resistant to clarithromycin: 4/96 (4.2%) were resistant at day 3 (acquired resistance) and 44/92 (47.8%) at day 14 (inducible resistance). Significant differences in clarithromycin-inducible resistance rates were observed among the 3 subspecies due to the different functionality of the *erm*(41) gene: MABSa (31/49, 63.3%) versus MABSb (13/13, 100%), *P* = 0.013; MABSa versus MABSm (0/30, 0.0%), *P* < 0.001; MABSb versus MABSm, *P* < 0.001.

We also found significant differences among subspecies in the rates of resistance to imipenem and tobramycin. Specifically, differences were found in the imipenem-resistant rates between MABSm (9/33, 27.3%) and MABSa (5/50, 10.0%), (*P* = 0.040) and between MABSm and MABSb (0/13, 0.0%), (*P* = 0.044). Tobramycin was ineffective against 81.3% of MABS isolates and significant differences were observed when comparing tobramycin-resistance rate of MABSa (33/50, 66.0%) versus MABSm (30/33, 90.9%), (*P* = 0.009). Although tobramycin-resistance rate of MABSb isolates (12/13, 92.3%) was higher than in MABSa isolates, statistical analysis did not show significant differences (*P* = 0.087), probably due to the small number of MABSb isolates.

### Genotypic susceptibility testing.

One out of 50 (2.0%) MABSa (named MABSa-61) and 3/33 (9.1%) MABSm isolates (named MABSm-19, MABSm-21, and MABSm-63) harbored mutations at positions 2058 or 2059 of the *rrl* gene and, thus, were resistant to clarithromycin (Table 2). All of them showed MICs > 16 μg/mL. None of MABSb showed acquired resistance to clarithromycin by the GenoType test.

**TABLE 2:** Genotypic and susceptibility testing results for clarithromycin and amikacin

Strains (N)^*a*^	GenoType results			Phenotypic resistance (MIC [μg/mL])	
	*erm* (41)	*rrl*	*rrs*	Clarithromycin	Amikacin
MABSa (31)	WT^*c*^	WT	WT	Inducible (8 to > 16)	S^*d*^ (4 to 16)
MABSa (17)	T28C	WT	WT	S (≤ 0.06 to 2)	S (2 to 16)
MABSa-70 (1)	T28C	WT	WT	S (0,5)	R^*e*^ (> 64)^*b*^
MABSa-61 (1)	WT	A2058C	WT	Acquired (> 16)	S (16)
MABSm (30)	WT	WT	WT	S (0.125 to 4)	S (≤ 1 to 16)
MABSm-19 (1)	WT	A2058G	A1408G	Acquired (> 16)	R (> 64)
MABSm-21 (1)	WT	A2058G	WT	Acquired (> 16)	S (4)
MABSm-63 (1)	WT	A2059C	WT	Acquired (> 16)	S (16)
MABSb (13)	WT	WT	WT	Inducible (> 16)	S (4 to 16)

a Names given internally: subspecies-number (N).

b Amikacin resistance not detected by the GenoType test. Mutations at different locus of *rrs* gene or other resistance mechanisms should be explored.

c WT: Wild-type;

d S: Susceptible;

e R: Resistant.

Regarding the *erm*(41) gene, 18/50 (36.0%) MABSa isolates harbored the T28C polymorphism and all MABSb strains showed the wild-type form of *erm*(41) gene. Genotypic results corresponded exactly with clarithromycin susceptibility phenotypes. All MABSb isolates showed inducible resistance to clarithromycin by the microdilution method and MABSa isolates harboring the T28C substitution were susceptible to clarithromycin.

As for amikacin resistance, 2/96 (2.1%) strains showed *in vitro* resistance, both with a MIC > 64 μg/mL. One of them belonged to the *massiliense* subspecies (MABSm-19) and the GenoType test revealed a mutation in the *rrs* gene. The other strain that presented phenotypic resistance to amikacin was a MABSa strain (MABSa-70). In this case, the GenoType test did not reveal the A1408G mutation in the *rrs* gene and showed the wild-type *rrs* band (Table 2).

Regarding clarithromycin and amikacin, overall agreement between GenoType and susceptibility testing methods for all the genes studied [*erm*(41), *rrl*, and *rrs*] was 99.0% (95/96).

## DISCUSSION

Herein, we investigated the epidemiological distribution of MABS subspecies as well as phenotypic and genotypic susceptibility profiles in a large number of clinical isolates. To the best of our knowledge, this is the first major epidemiological study of MABS in Madrid, and it may help to clarify the epidemiology of this emerging pathogen in our region.

In the last decades, an increase in NTM prevalence has been described worldwide, although the most prevalent species vary depending on the geographical area studied (19). Hoefsloot et al. conducted a study including NTM isolates from more than 20 000 patients from 30 countries and concluded that M. abscessus was one of the most frequently isolated rapid-growing mycobacteria (20). Accordingly, in a recent multicenter study, we found a 2-fold increase in MABS isolates from 2013 to 2017 in Madrid (3). This observation prompted us to carry out the present study to better understand the epidemiology of MABS in our region. Our results show that the upward trend of MABS isolates continued during the 2016 to 2019 period. However, in 2020, we observed an important decrease in the number of isolates reported, probably as a result of the impact that the COVID-19 pandemic had on health care systems.

It is known that MABS subspecies distribution also varies within geography, even between regions of the same country. In addition, since there is still controversy in the taxonomy of this pathogen, differences can be found in the reported prevalence depending on the nomenclature used in each study. Although there are studies from East Asia (21–23) that report MABSm as the more prevalent subspecies, MABSa remains the subspecies most frequently reported globally, followed by MABSm, and reporting of MABSb remains relatively unusual (24–26). Accordingly, we also found that MABSa and MABSm were the most prevalent subspecies (52.1% and 34.4%, respectively) in our region. Unexpectedly, we found a relatively higher rate of MABSb, as reported in other studies conducted in Europe (1, 27, 28).

In line with most of the previous studies, we have observed that MABS isolates circulating in our region showed high *in vitro* resistance rates (> 80%) to doxycycline, fluroquinolones (ciprofloxacin and moxifloxacin), and/or cotrimoxazole. The resistance rate to tobramycin was 81.3% in this study, but this percentage varied according to the subspecies. In fact, MABSa showed a lower rate of resistance to tobramycin compared to MABSb and MABSm. These differences have also been reported in a study conducted in Shanghai, China (29).

Among the most effective antimicrobials *in vitro*, remarkable findings concern imipenem, amikacin, and clarithromycin. In the present study, imipenem resistance was found in less than 15% of the isolates, which is similar to that reported by Guo et al. in 2021 (29), but notably lower than resistant rates reported in most studies, ranging from 40% to 98% (22, 30, 31). As previously reported (21), we found that imipenem resistance rate of MABSm was higher than that of MABSa and MABSb, being the difference statistically significant. Regarding tigecycline, it has demonstrated strong *in vitro* activity against rapidly growing mycobacteria and has already been recommended to be used in the treatment of M. abscessus disease according to the current guidelines (32, 33). Although no susceptibility breakpoint has been established yet for tigecycline, we found that tigecycline displayed excellent *in vitro* activity against all but 1 isolate (MIC = 8 μg/mL), with MICs not exceeding 1 μg/mL.

Several previous studies have reported low rates of amikacin-resistant MABS (< 6%) (22, 23, 29, 30). Consistent with this, we only found 2 MABS isolates (2.1%) resistant to amikacin. In 1 of these 2 isolates, the Genotype test revealed the A1408G mutation in the *rrs* gene. The other isolate did not show any mutation and presented hybridization in the wild-type band of the *rrs* gene. Although the most common resistance mechanisms to amikacin can be detected by this test, other mechanisms have been described (34). Therefore, further analysis is needed to elucidate the resistance mechanism to amikacin of this isolate.

In our study, genotypic results corresponded exactly with clarithromycin susceptibility phenotypes. All MABS isolates (1 MABSa and 3 MABSm) resistant by broth microdilution at day 3 showed mutations in the *rrl* gene by the Genotype test and none of the MABSm isolates exhibited inducible resistance (at day 14 of incubation), which is consistent with the presence of a nonfunctional copy of the *erm*(41) gene in this subspecies (13). Finally, phenotypic inducible clarithromycin resistance was detected in MABSa and MABSb isolates harboring the wild-type *erm*(41) gene. Only those associated with a T28C polymorphism were found to be nonfunctional and showed susceptibility to clarithromycin. Other studies have also reported good correlation between phenotypic resistance to clarithromycin and the associated genotypes determined by the Genotype test (18).

Overall, half of the MABS strains showed resistance (inducible or acquired) to clarithromycin. This is a worrisome fact given that clarithromycin has shown the greatest correlation between *in vivo* and *in vitro* results (35), and poorer clinical outcomes have been evidenced in patients infected with macrolides resistant MABS (36). Nonetheless, 36.0% of the MABSa isolates in our study harbored the T28C mutation in the *erm*(41) gene and were therefore clarithromycin-susceptible at day 14 in the absence of other resistance mechanisms. Importantly, the proportion of susceptible MABSa isolates that we report is higher than in other studies, where only 4% to 17% of MABSa isolates had the T28C polymorphism (23, 24, 37).

The results reported herein should be considered in the light of some limitations. Due to the retrospective nature of the study, we were unable to obtain data related to previous antimicrobial treatments, which may play a role in the resistance profile and the selection of resistance in some of the clinical isolates. Since the strains were not sequenced, we cannot ensure the correct identification of the subspecies. However, as results referred to the *erm*(41) gene (which differs between subspecies) were consistent with the phenotypic profile, nothing makes us suspect misidentification at the subspecies level.

To summarize, MABSa was the most frequently isolated subspecies during the study period in our region. Although MABS is known to be resistant to most antibiotics, we observed a high proportion of MABS strains showing *in vitro* susceptibility to amikacin, linezolid, cefoxitin, and imipenem; however, more studies are needed to correctly establish the *in vitro-in vivo* correlation. Half of the MABS isolates were resistant to clarithromycin (whether acquired or inducible), with resistance rates differing between subspecies. Finally, Genotype serves as a reliable tool for detecting mutations predictive of antimicrobial resistance in MABS.

## MATERIALS AND METHODS

Following approval from our Local Institutional Review Board, we performed a retrospective analysis from 2016 to 2021 of all MABS clinical isolates collected from 7 tertiary hospitals located in Madrid. Clinical and microbiological information of each episode was extracted from (electronic) databases. To avoid bias in our retrospective analysis, only the first isolation per patient was included in the study. In addition, demographic information was collected including gender, age, and nationality.

### Clinical isolates.

M. abscessus isolates’ first identification to the species level was performed using the GenoType CM test (Hain LiveScience). All isolates were kept frozen at −20°C. After recovering from storage, they were seeded in mycobacterial growth indicator tube (MGIT) liquid medium (Becton, Dickinson and Company, Franklin liquid medium Lakes) and incubated at 37°C. After bacterial growth, genotypic and phenotypic drug susceptibility tests were carried out.

### Phenotypic susceptibility testing.

Phenotypic antimicrobial resistance was studied by broth microdilution method using the RAPMYCOI Sensititre titration plates (Thermo Fisher Scientific) following the Clinical and Laboratory Standards Institute (CLSI) standard M24 for susceptibility testing (38). The following antibiotics were tested: cotrimoxazole, ciprofloxacin, moxifloxacin, cefoxitin, amikacin, doxycycline, tigecycline, clarithromycin, linezolid, imipenem, and tobramycin. MICs were determined after 3 days of incubation, except for clarithromycin, in which the incubation period was extended to 14 days. MICs were interpreted as resistant, susceptible with increased exposure, or susceptible following the CLSI recommendations (39).

### Genotypic susceptibility testing.

GenoLyse DNA extraction kit and GenoType Mycobacterium NTM-DR test (Hain LiveScience) were used to characterize M. abscessus subspecies and to determine the presence of mutations in macrolides-resistance associated genes [*erm*(41), *rrl*] and aminoglycosides (*rrs*). All steps were performed according to manufacturer’s instructions.

The assay was based on the DNA-STRIP technology, consisting of DNA extraction followed by multiplex amplification and reverse hybridization. Each strip contained different probes targeting each gene, therefore being capable of detecting the most common mutations related to macrolides and aminoglycosides resistance. The probes were designed to detect: the T28C substitution at the *erm*(41) gene, 4 point mutations at the *rrl* gene (A2058C/G, A2059C/G), and 1 mutation at the *rrs* gene (A1408G).

### Statistical analysis.

Qualitative variables were presented with their frequency distribution (gender, nationality, and clinical sample). Quantitative variables were shown with its mean value and standard deviation (SD).

The association between qualitative variables (subspecies versus antimicrobial resistance) was evaluated with the Chi-square χ^2^ test or with the Fisher's exact test when more of 25% of the expected values were less than 5.

A significant value of 5% was accepted for all tests. Data analysis and processing were performed with IBM SPSS Statistics v.26 software.
